# Factors affecting the intention to use COVID-19 contact tracing application “StaySafe PH”: Integrating protection motivation theory, UTAUT2, and system usability theory

**DOI:** 10.1371/journal.pone.0306701

**Published:** 2024-08-01

**Authors:** Ardvin Kester S. Ong, Yogi Tri Prasetyo, Regina Pia Krizzia M. Tapiceria, Reny Nadlifatin, Ma. Janice J. Gumasing

**Affiliations:** 1 School of Industrial Engineering and Engineering Management, Mapúa University, Manila, Philippines; 2 E.T. Yuchengo School of Businseeess, Mapúa University, Makati, Philippines; 3 International Bachelor Program in Engineering, Yuan Ze University, Chung-Li, Taiwan; 4 Department of Industrial Engineering and Management, Yuan Ze University, Chung-Li, Taiwan; 5 Department of Information Systems, Institut Teknologi Sepuluh Nopember, Kampus ITS Sukolilo, Surabaya, Indonesia; 6 Department of Industrial and Systems Engineering, Gokongwei College of Engineering, De La Salle University, Manila, Philippines; University of Central Punjab, PAKISTAN

## Abstract

**Purpose:**

StaySafe PH is the Philippines’ official contact tracing software for controlling the propagation of COVID-19 and promoting a uniform contact tracing strategy. The StaySafe PH has various features such as a social distancing system, LGU heat map and response system, real-time monitoring, graphs, infographics, and the primary purpose, which is a contact tracing system. This application is mandatory in establishments such as fast-food restaurants, banks, and malls.

**Objective and methodology:**

The purpose of this research was to determine the country’s willingness to utilize StaySafe PH. Specifically, this study utilized 12 latent variables from the integrated Protection Motivation Theory (PMT), Unified Theory of Acceptance and Use of Technology (UTAUT2), and System Usability Scale (SUS). Data from 646 respondents in the Philippines were employed through Structural Equation Modelling (SEM), Deep Learning Neural Network (DLNN), and SUS.

**Results:**

Utilizing the SEM, it is found that understanding the COVID-19 vaccine, understanding the COVID-19 Delta variant, perceived vulnerability, perceived severity, performance expectancy, social influence, hedonic motivation, behavioral intention, actual use, and the system usability scale are major determinants of intent to utilize the application. Understanding of the COVID-19 Delta Variant was found to be the most important factor by DLNN, which is congruent with the results of SEM. The SUS score of the application is "D", which implies that the application has poor usability.

**Implications:**

It could be implicated that large concerns stem from the trust issues on privacy, data security, and overall consent in the information needed. This is one area that should be promoted. That is, how the data is stored and kept, utilized, and covered by the system, how the assurance could be provided among consumers, and how the government would manage the information obtained. Building the trust is crucial on the development and deployment of these types of technology. The results in this study can also suggest that individuals in the Philippines expected and were certain that vaccination would help them not contract the virus and thus not be vulnerable, leading to a positive actual use of the application.

**Novelty:**

The current study considered encompassing health-related behaviors using the PMT, integrating with the technology acceptance model, UTAUT2; as well as usability perspective using the SUS. This study was the first one to evaluate and assess a contact tracing application in the Philippines, as well as integrate the frameworks to provide a holistic measurement.

## 1. Introduction

One of the most important aspects of pandemic preparedness was the COVID-19 traceability of contacts [1, 2]. It is an approach to avert the situation of COVID-19 by tracing and tracking all of the potential individuals in close contact with the COVID-19 patient/suspect [3–5]. Contact tracing was a vital part of the resilience of a country, especially during the Delta variant [6]. This variant was more contagious and dangerous as many people were hospitalized and were more susceptible to vaccines than others [6]. With recent developments, contact tracing could assist to reduce transmissions and secondary illnesses, particularly amid the Delta variant outbreak [7]. Moreover, its capability to monitor and consider positive health promotion among individuals was vital. Therefore, the need for continued development and utility for future use is needed. One of the most widely utilized contact tracing platforms is mobile-based COVID-19 contact tracing.

Mobile-based COVID-19 contact tracing has been utilized by many countries, especially during the Delta period. The NZ COVID Tracer application was used in the first example of the Delta variation found in New Zealand [8]. The data acquired helps them identify the close contacts of a person and the places the individual went. Moreover, it helped the contact tracers save time and effort in acquiring essential data that could aid in controlling the pandemic [9, 10]. The United Arab Emirates (UAE) has also utilized the Al Hosn application as its official contact tracing application, which was proven to lessen contact of the virus [9, 11]. As evidenced by the study of Khan et al. [12], it was favored by individuals living in the country. It was seen that positive response from social media posts were evident. Even among social media figures, the contact tracing application gained positive insights. In Southeast Asia, Singapore uses TraceTogether as its official contact tracing application [13]. Chow et al. [14] explained that this built a new passage for future health monitoring. Moreover, another COVID-19 contact tracing application in Southeast Asia is the StaySafe PH in the Philippines.

StaySafe PH is the Philippines’ primary contact tracing program for controlling the propagation of COVID-19 and promoting a uniform contact tracing system [15]. The application was used by 15 million users and was utilized widely by 700 Local Government Units (LGUs) [15, 16]. As the COVID-19 Delta variant has reached and continues to spread in Metro Manila, the LGUs imposed a strict digital contact tracing and a faster vaccine inoculation that would aid in the avoidance of spreading the variant further and also prepare for the other variants that could come [17, 18]. However, the official government contact tracing application during the peak of the Delta outbreak has received backlash from users [19]. They deemed the application useless as it only has minimal effect in controlling the virus, and the developers should improve the features more to cater to the needs and wants of the users. Moreover, implications from the Filipinos indicated that it may be a creeping surveillance among private information [20] leading to huge trust issues, utility, and misconception. Only one study was seen on the positive and effective perception of the application, found in the provinces of the country [21]. This highlights a crucial need to develop if not redevelop, promote, and recognize several implications for the enhanced utility of the contact tracing application–especially for future use.

The StaySafe PH has various features such as a social distancing system, LGU heat map and response system, real-time monitoring, graphs, infographics, and its primary purpose as a contact tracing system [22]. This application is mandatory in establishments such as fast-food restaurants, banks, and malls during the pandemic [23]. To which, the customers will be required to scan a QR code provided by the establishment that will enable them to answer questions regarding their personal information and their symptoms, if there are any [23]. The analyzed health reports based on the information provided by the users will be passed to LGUs to trace those they suspect to be positive and their close contacts [24]. The application will utilize the Google Apple Exposure Notification System (GAEN) to notify Android and iOS users of any exposure near positive cases [25]. It was said that if everyone can consent to digital contact tracing, the exposure detection mechanism may be considered successful. These developed technologies have presented significant help and development, especially in mitigation of the virus spread during the pandemic [26]. Thus, the need for assessment is needed since current threats are evolving with the recent news of new respiratory illnesses spreading [27].

Based on related studies covering contact tracing applications and other developments, the current study considered encompassing health-related behaviors using the PMT, integrating with the technology acceptance model, UTAUT2; as well as usability perspective using the SUS. This study was the first one to evaluate and assess a contact tracing application in the Philippines, as well as integrate the frameworks to provide a holistic measurement. The analysis and constructs of this study can be the basis of succeeding studies that may deal with a contact tracing application. Identifying the elements that influence the intent to utilize the application will help understand how Filipinos perceive the official government contact tracing application, as well as how to make it more effective for the people to utilize.

Moreover, the analysis of this study will also help improve the application in accordance with the users’ requirements and expectations to reduce negative feedback, which can be applied even in other countries. Furthermore, the analysis can be utilized as a theoretical foundation in assessing the initiative to employ a contact tracing program on a global scale. The suggested implications based on the findings of this study could further develop these types of technology and systems, especially in future events where health crises arise again.

## 2. Related studies and conceptual framework

### 2.1 Literature review

Previous studies have assessed various digital contact tracing applications amid the COVID-19 Pandemic. In the study by Li et al. [28], researchers utilized linear regression analysis and mediation analysis to determine that individual variations exert a greater influence on the adoption and intent of employing contact tracking programs than the application’s design, which has a lesser influence. Mouter et al. [29] study utilized descriptive analysis, mixed logic model, and simulated maximum likelihood methods. The researchers identified that individuals would most likely install digital contact tracing applications with a high societal impact [29]. Chen et al. [30] utilized Partial Least-Squares Structural Equation Modelling. The study aims to identify the reasons for the hesitancy of individuals in using contact tracing applications. The researchers deduced that cognitive trust affects the individuals’ willingness to use contact tracing applications by integrating the Perception-Trust-Behavior Intention framework.

Strong theoretical frameworks were utilized in several studies. Privacy calculus Theory and Social Exchange Theory were integrated by Fox et al. [31] to know the different influences regarding the privacy concerns of individuals and the benefits of acceptance of contract tracing applications to improve the adoption rate. Privacy concerns need to be addressed as they always negatively affect the acceptance of people with contact tracing applications [31]. Based from the study of Trkman et al. [32], The Crisis Decision Theory was applied to analyze the public and personal advantages of using proximity monitoring apps. Their objective was to comprehend the link among the perceived intensity of the crisis as well as the behavioral intent of using the applications. It was expressed that individuals are most comfortable using proximity tracing applications as preventive behavior if they know the seriousness of the crisis.

Public Value Theory integrated by Gerli et al. [33] was utilized to analyze the adoption of eHealth applications. Exogenous factors such as the ‘digital divide’ influenced some people to have no access to the technology, so contact tracing would not be as effective since only limited people can participate in contact tracing. Guillon and Kergall [34] used the Health Belief Model to evaluate the use of contact monitoring apps. According to the investigation, consumers are more inclined to utilize contact monitoring apps due to the anticipated threat of a pandemic, the anticipated advantages of confidence in the authorities, and risk preferences. The UTAUT2 with Public Value and Trust Concept was utilized by Syamsudin et al. [35] to identify the behavioral intention of using e-government services. Their study concluded that public value, habit, and effort expectancy positively influenced the choice to engage with digital government services. Regardless of the studies using various frameworks, some previous studies have also applied Protection Motivation Theory, UTAUT2, and even SUS in assessing digital contact tracing applications.

Protection Motivation Theory is a widely used empirical model that explains why and how individuals behave when protecting their health [36]. Individuals are said to have mediating experiences such as threat and coping appraisal whenever they are in a particular environment and surrounded by intrapersonal factors [36, 37]. The study of Sharma et al. [33] aims to find the individual’s objectives for adopting digital contact tracing applications. They used various theories such as Procedural Fairness Theory, Dual Calculus Theory, Protection Motivation Theory, Theory of Planned Behavior, and Hofstede’s Cultural Dimension Theory. The researchers were able to deduce that the perceived vulnerability positively influences the individual’s adoption while the apparent efficacy of confidentiality regulations has a negative impact.

UTAUT2 is an empirical and conceptual study introduced in the year 2012 due to the increasing usage of technology. It is the extended version of UTAUT that includes hedonic motivation, habit, and price value [38]. Several studies have used UTAUT and UTAUT2 in investigating technology-related studies. Walrave et al. [39] used UTAUT to identify the elements that affected a person’s intention to utilize a contact tracing application. According to the findings, an important predictor is performance expectancy, subsequent to facilitating conditions and social influence. Effort Expectancy has minimal to no effect on the intention. Duarte and Pinho [40] used UTAUT2 to identify which factors of UTAUT2 would significantly impact the individuals’ willingness to use mobile health. According to the findings, performance expectation was the strongest indicator of mobile health uptake. Alam et al. [41] evaluated and observed the users of mobile health services regarding their intent in employing the application and assessed their use patterns during self-imposed quarantine to further analyze the individuals’ psychological state by UTAUT2. Performance expectancy, effort expectancy, social influence, facilitating condition, and hedonic motivation were the main determinants of mobile health applications’ usage intention.

Aside from the theoretical foundations, studies have also considered the utilization of predictive methodologies that have been found to have higher and more accurate output. Xie et al. [42] have utilized the Bayesian network structure for an interpretative structural model (ISM-K2) hybrid. Their study opted to predict the effectiveness of vaccines in China. From their study, historical data and expert judgement were utilized. However, Zheng et al. [43] implied natural language processing using deep learning. A positive result was seen based on the improved accuracy, predictive output, and ability to transformation of generative dialogue for Chinese texts. It was seen that deep learning processes provided a more cohesive analysis, relating to a higher accuracy output. As support, the study of Li et al. [44] presented how the recent trend of analysis delved deeper into machine learning tools as deemed more appropriate. This power tool has been widely considered and is suggested to be considered by related studies. Presented in their sophisticated review were patterns, proof, and utility of powerful machine learning algorithms when assessing smartphone application usage.

### 2.2 Conceptual framework

**Fig 1:**
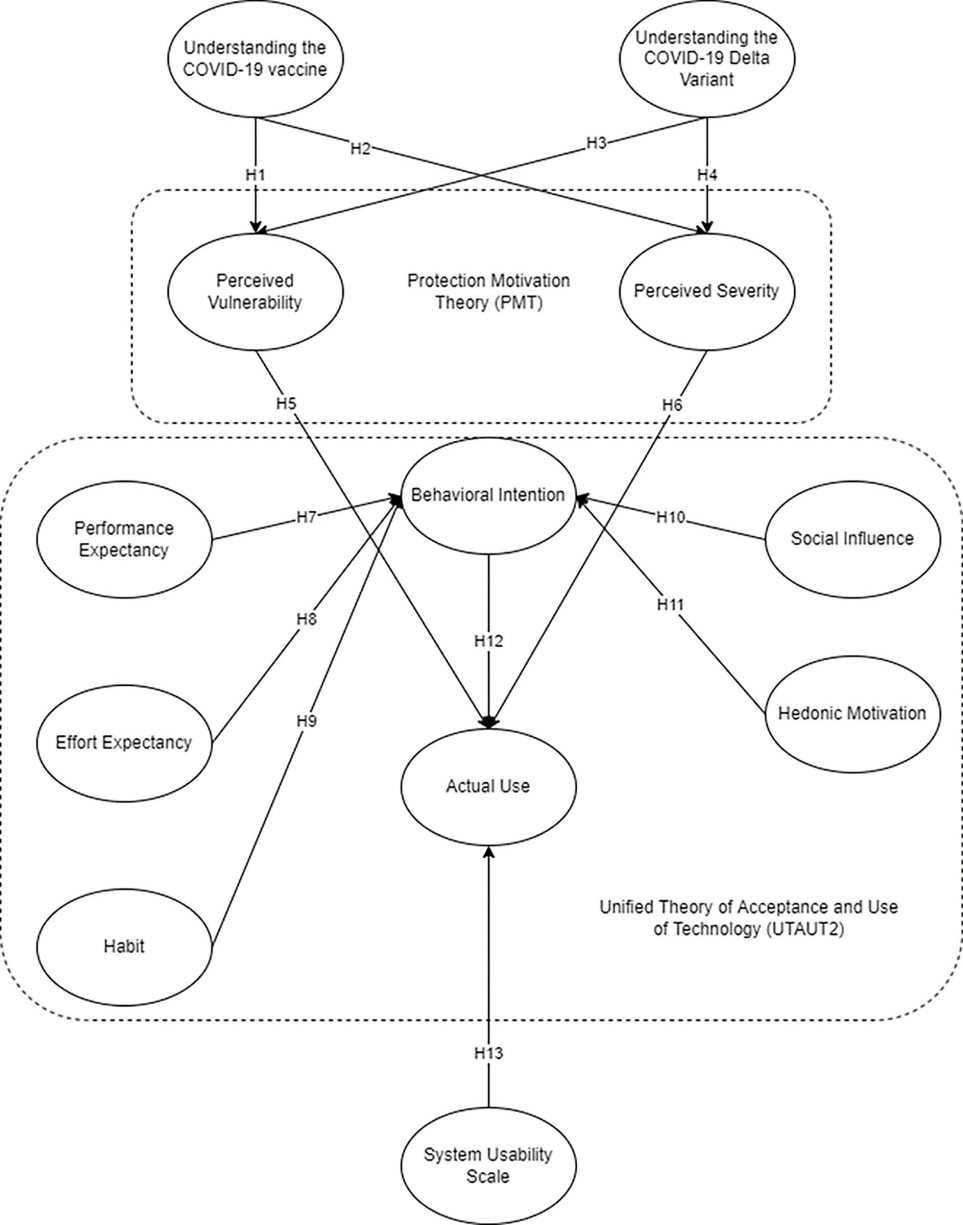
Conceptual framework.

The conceptual framework in Fig 1 integrated varying models such as PMT, UTAUT2, and SUS. The framework assessed the intention to use the StaySafe application by evaluating the user’s prior knowledge of the pandemic and its safety precautions, the perceived threat of the pandemic to users’ health, the adoption for fresh advances in technology, as well as the application’s usefulness.

The variables under the PMT are Perceived Vulnerability and Perceived Severity. The PMT is employed in the framework as it effectively forecasts the adoption of new protective technologies [45]. The StaySafe contact tracing application is considered a protective technology that helps users avoid COVID-19 suspects. Thus, in the framework, the PMT variables are depicted to analyze further the effect it has on the respondents’ actual use of the application.

On the other hand, Performance Expectancy, Effort Expectancy, Social Influence, Hedonic Motivation, Habit, Behavioral Intention, and Actual Use are the variables from the UTAUT2. The model assesses the adoption of the consumers to new technology [46]. The UTAUT2 model is appropriate in the contact tracing application context as StaySafe PH is an innovative technology created during and for the worsening situation of the pandemic, and the application’s primary consumers are the people residing in the Philippines.

In addition, SUS is also a part of the model. It is a reliable evaluation theory that assesses any technology based on its usability and ease of use from its users’ perspective [47]. The model is appropriate in the framework because it can quickly evaluate the respondents’ view of the contact tracing application and how easy and usable it is to navigate.

Understanding the COVID-19 Vaccine and Understanding the COVID-19 Delta Variant are the factors that the authors included in the framework. It aims to know the prior knowledge of the respondents with regards to the Delta Variant and COVID-19 vaccines since the purpose of the contact tracing application is to avert the situation of COVID-19 and aid in protecting the people. As a reflection, the PMT is enclosed in the top and first box in Fig 1.

As explained under the PMT, whenever unexpected events that expose individuals to risk their health occur, such as the pandemic, they are most likely to predicate threat and coping appraisal as predictors of their health behaviors [48]. Events that would compromise their health will motivate individuals to protect themselves. Perceived vulnerability is one of the determinants that can drive individuals to cooperate with the public in adopting health-protective behaviors [49, 50]. It was explained that those who feel they are more vulnerable to the infection are the ones who adopt more protective measures to protect themselves than those who believe that they are less susceptible [50]. Obtaining COVID-19 vaccines is a preventive measure that individuals can take to reduce the worry about the infectious disease [51]. It is posited that adequate knowledge and understanding of the COVID-19 vaccine will help people be more engaged in getting one to protect themselves. Thus, it would make them less susceptible to risks to their health. Hence, the researchers hypothesized that:

**H1:** Understanding the COVID-19 vaccine has a direct effect on Perceived Vulnerability.

The perceived severity is the magnitude of the consequences if the person’s health is at risk [37, 52]. According to Weinstein and Klein [52], when it considers health behavior, it is believed that an individual who thinks that he/she is experiencing perceived risk is also more inclined to have an increase in precautionary behavior. Thus, the researchers anticipated that recognizing the COVID-19 vaccination as a preventative intervention might have a direct impact on people’s perceptions of severity.

**H2:** Understanding the COVID-19 vaccine has a direct effect on Perceived Severity.

In the study of Boyraz et al. [53], those people who were aware of their vulnerability to COVID-19 were the ones who felt that COVID-19 is fatal and considered to be life-threatening for them, thus leading them to protective behavior. Delta variants are known to be highly transmissible and deemed to be fatal, making them more dangerous for the vulnerable ones [54]. Accurate information and being knowledgeable about the preventive measures regarding COVID-19 will help in lessening the threat of vulnerability. Thus, the researchers hypothesized that:

**H3:** Understanding the COVID-19 Delta variant has a direct effect on Perceived Vulnerability.

The perceived severity influences how the individuals would perceive the information acquired regarding COVID-19 [55]. A minimal understanding and knowledge of COVID-19 could increase the risk of transmitting the disease, thus making it difficult to control [56]. In the study of Makhanova and Shepherd [57], it was explained that people need to cooperate with preventive behaviors to control the pandemic. However, some people have not experienced such threats within their environment, thus belittling the preventive behaviors that individuals need to do. In this case, the researcher hypothesized that:

**H4:** Understanding the COVID-19 Delta variant has a direct effect on Perceived Severity.

In the study by Mamra et al. [58], they proposed using both PMT and UTAUT2 to assess user acceptance of health records. The perceived threats and vulnerability constructs measured an individual’s capacity toward dealing with the repercussions of the pandemic. It has been shown to influence people’s adoption of new technologies. Their study proposed that the integration of both theories provided a more coherent analysis. Moreover, Behavioral Intention has been proven to be the immediate indicator of actual usage [59]. Thus, it is possible that the users would be more likely to undergo actual usage behavior right away than having to go through the conative stage if they perceived the application of technology to be necessary for protecting themselves [41, 59]. In this study, as people recognize the virus’s vulnerability and dangers, individuals would consequently be more likely to utilize the application to help protect themselves from the virus. Therefore, the researchers derived the following hypotheses:

**H5:** Perceived Vulnerability has a direct effect on Actual Use.
**H6:** Perceived Severity has a direct effect on Actual Use.

Walrave et al. [39] stated that performance expectancy is the benefits that users expect whenever they use the application. In the study, they expected that the users would most likely intend to install and use the application as this will help prevent the virus and detect positive cases. The performance expectancy is a vital construct for various research on the widespread utilization of software applications [39, 40]. Moreover, individuals have a greater likelihood to download and utilize a program convenient for them, especially if it needs less effort in using it while meeting their needs [40]. Under this situation, consumers will utilize the program if they believe it is simple to use [39]. For this reason, the researchers hypothesized that:

**H7:** Performance Expectancy has a positive influence on Behavioral Intention.
**H8:** Effort Expectancy has a positive influence on Behavioral Intention.

According to Syamsudin et al. [35], the group where an individual belong can change the individual’s perspective if they have the same purpose in using the technology. Multiple investigations have found that societal bearing has a favorable impact on users’ behavioral intentions [60]. However, Walrave et al. [39] stated that the role of societal bearings degrades whenever the individual has enough experience in using the technology. Individuals will intend to use the application if the people support and persuade them to utilize the application. Thus, the researchers hypothesized:

**H9:** Social Influence has a positive influence on Behavioral Intention.

Hedonic motivation is the construct pertaining to the enjoyment of using the technology. The individual would be most likely to utilize the application if they deemed it fun and pleasurable to employ [61]. The investigation by Alam et al. [41] stated that hedonic motivation is a strong predictor of decision-making intent adhering health apps for mobile devices. Individuals who have a higher level of hedonic drive for cutting-edge innovations are more probable to install and utilize the application [62]. Based on the studies, the researchers hypothesized that:

**H10:** Hedonic Motivation has a positive influence on Behavioral Intention.

According to Vinnik [61], mobile applications are meant to be used daily, which would create a habit for the users. Relating to the constant use of contact tracing applications, it could be posited that habit has been established since the requirement to constantly utilize this has been administered. Especially in the Philippines where every transaction or action needs the scanning of contact tracing applications, people have gained the habit of considering the application ready for every activity. To which, habit is also one of the strong determinants [35]. It was added that a positive habit results in loyalty and satisfaction among users. As the user acquires habit, it would be arduous for them to leave and uninstall the application. Consequently, an individuals’ behavioral motivations have been found to be an immediate indicator of their actual usage pattern when using mobile applications [41]. It was stated in the same study that individuals are coherent to transform from their behavioral intention stage, also known as the conative stage, to their actual behavior or action stage. According to Gansser and Reich [62], behavioral intention strongly relates to actual user behavior. Hence, the researchers hypothesized that:

**H11:** Habit has a positive influence on Behavioral Intention.
**H12:** Behavioral Intention has a direct effect on Actual Use.

SUS is a human-computer interaction approach used widely to evaluate the usability of new technology [63]. The SUS is highly reliable in evaluating the perceived usability and is being employed to evaluate the functionality of the majority of apps for mobile devices [63, 64]. If the application is usable, more individuals will install and utilize the application. Hence, it was hypothesized that:

**H13:** System Usability Scale has a direct effect on Actual Use.

## 3. Methodology

This study was approved by Mapua University Research Ethics Committees (FM-RC 21–77). A signed online consent form was also collected from each participant prior to the questionnaire data collection.

### 3.1 Respondents

The survey was open to people living in the Philippines from July 1^st^, 2021 to September 30^th^, 2021. Due to the rigorous lockdown enforcement in the Philippines, this survey was delivered online using convenience sampling. The response acquired a total of 646 respondents (S1 Table) which mostly made up of individuals ages 15 to 24 years old (47.21%), followed by 25 to 34 years old (21.83%), and 35 to 44 years old (16.87%). To which, the Female (56.67%) and Male (43.34%) respondents majority lived in Quezon City (37.00%), Paranaque City (9.29%), and Manila City (4.80%). The respondents comprised 26.32% which are Secondary Graduate and 50.46% are bachelor’s degree Holder that has below Php15,000 (44.27%) or Php 15,001-Php 30,000 allowance/monthly income. Moreover, 44.12% of the respondents are not enrolled in PhilHealth, while most of the respondents are enrolled with 55.88%. The majority of the respondents use the StaySafe application (68.73%); however, there are still numerous who have not used the application (31.27%).

### 3.2 Questionnaire

The internet-based questionnaire was conducted using a 5-point Likert Scale. The participants were given the prospect of selecting Strongly Disagree, Disagree, Neutral, Agree, or Strongly Agree, depending on their level of agreement in every statement. The questionnaire consisted of 13 parts (S1–S3 Tables), adapted from related studies [25, 35, 42, 50, 58, 59, 62–74]. The survey included (1) Demographics, (2) Understanding the COVID-19 Vaccine, (3) Understanding the COVID-19 Delta Variant, (4) Perceived Vulnerability, (5) Perceived Severity, (6) Performance Expectancy, (7) Effort Expectancy, (8) Social Influence, (9) Hedonic Motivation, (10) Habit, (11) Behavioral Intention, (12) Actual Use, and (13) System Usability Scale.

### 3.3 Structural Equation Modeling

Structural Equation Modelling (SEM) was used in the study for assessing intentions to use StaySafe PH among Filipinos. As explained by Dash and Paul [65], covariance-based SEM is widely known and utilized for established framework analysis. In relation to this study, the integrated framework considered was from established PMT, UTAUT2, and the system usability theory. The analysis, as a reflection from Dash and Paul [65], involved the identification of a causal relationship affecting the target output. In addition, measure item assessment could be performed to assess the unobserved variables considered in this study. It was suggested that newly developed framework (may it be through integration of extension) could consider SEM analysis for better and accurate insights [65, 66]. Hair [66] also established this capability among SEM analyses. In accordance, this study considered the validity of the model, interrelationships among latent variables, and measure item validity.

### 3.4 Deep Learning Neural Network

The survey acquired a total of 646 respondents as valid data in estimating the elements influencing the intent of Filipinos to utilize StaySafe PH. The valid data presented a total of 40,698 data points coming from 646 valid responses and 63 measure items. This was considered for the deep learning algorithm to represent the target objectives and input variables. Prior to which, data pre-processing was done to run the Deep Learning Neural Network (DLNN). Utilizing SPSS 25, it was seen that there were no missing data. To which, correlation analysis was utilized for the data-cleaning process. Non-significant latent with a p-value greater than 0.05 and correlation value less than 0.20 were removed [67]. Based on the correlation result, two indicators from the Perceived Vulnerability were eliminated, and one indicator from the Habit resulting in a decrease in indicators from 65 to 62 indicators. Thus, 62 indicators were considered significant for the machine learning algorithm (MLA) optimization.

DLNN is known in artificial intelligence and is a widely used type of MLA for the classification of supervised data [68, 69]. The DLNN model has immensely helped establish relationships between variables and extract necessary information from its input variables. A DLNN consists of an input layer in which the input data is being received, processed in several the hidden layer, and proceeds to the output layer where the prediction is formulated [68–70]. The DLNN approach is also being utilized in diverse fields such as speech and image recognition, forecasting of trends (*i*.*e*., economy and stocks), and behavior predictions. This was also incorporated in some studies to analyze further different information [71–73]. Neural networks (NN) such as DLNN have been used as the methodology of several studies alongside SEM. It has been revealed that the NN is an excellent MLA tool to establish a more accurate result and to understand further the results of the studies [74–77]. This is because Fan et al. [78] indicated that SEM alone would not be sufficient to predict the significant effect of factors due to the causal relationship present, and the indirect effect from one latent to another may have errors especially if relationships are far from each other.

Following the data cleaning process, data aggregation using mean was utilized for the initial optimization. Eleven latent variables were considered (UDV, UV, PV, PS, PE, EE, SI, HM, HB, and BI) as the input layer to predict factors affecting the use of the StaySafe PH contact tracing application among Filipinos. Data normalization using min_max scalar was employed utilizing Python 3.8 before running the DLNN. Moreover, this study used various combinations of activation functions (AF) and optimizers. The AF and the optimizer were determined by trying out the different combinations. Three AF for the hidden layer were considered: Tanh [79–81], Sigmoid [82–84], and Swish [85, 86]. In addition, there were two AF considered for the output layer: Sigmoid [79, 81, 83, 84, 87] and Softmax [87–89]. Lastly, three optimizers were also used for the DLNN: Adam [90–92], RMSProp [87, 93, 94], and SGD [95–97].

### 3.5 System Usability Scale

The SUS Score was utilized in this study to evaluate the SUS regarding the StaySafe PH application and further understand how usable the application is among Filipinos regarding usability and learnability. The SUS score was based on the ten-item questionnaire to assess the application’s usability. The interpretation, acquired from the study of Ayoub [98], was utilized to assess this study.

Different from several studies that utilized SUS [47, 99, 100], this study transposed the negative statements in the even numbers into positive statements. For instance, in standard SUS, question number 2 should be “I found the StaySafe application unnecessarily complex.” and was changed into “I found the StaySafe application not complex” in this study. The reason for which is that the SUS as one endogenous latent variable was set in this study to holistically assess the actual use of StaySafe PH. In SEM, all statements under one latent variable should be in the same direction, either all positive or all negative. On the other hand, in calculating the SUS score for StaySafe Philippines, questions in even numbers were re-transposed to the original negative statements to calculate the actual score.

## 4. Results

### 4.1 Structural Equation Modeling

**Fig 2:**
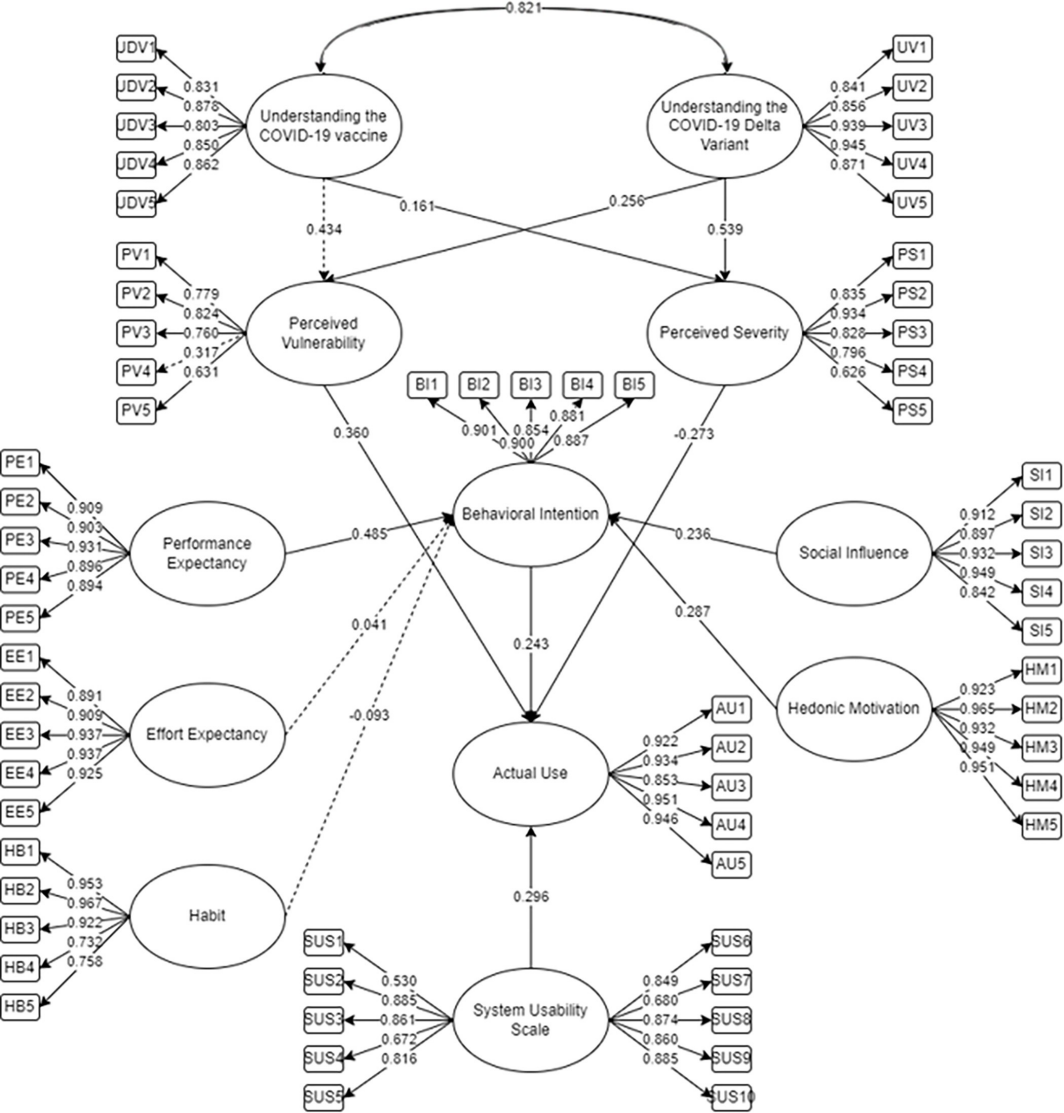
The initial SEM for the intention to use StaySafe PH.

**Fig 3:**
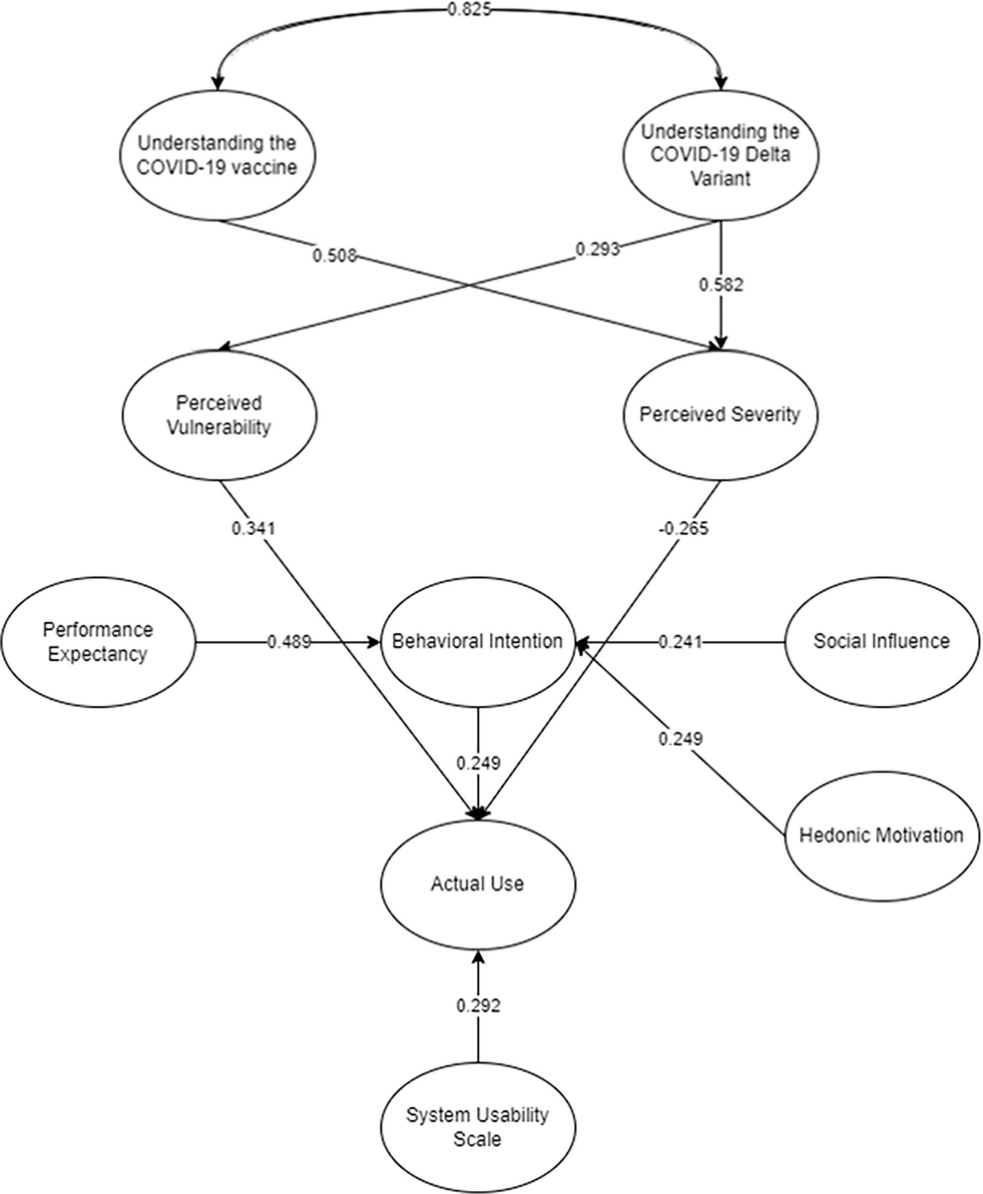
The final SEM for the intention to use StaySafe PH.

The SEM was utilized in AMOS 25 and SPSS 25 to gather the following initial results (Fig 2). Following the suggestion of Hair [66], relationships should have p-values less than 0.05 to be considered significant. In accordance, the AMOS output for the measure items should be greater than 0.50 to be considered significant [68]. As presented in the initial SEM (Fig 2), all relationships and items not within the suggested threshold are reflected as broken lines. It was further suggested that the removal of these should be performed to present the final SEM (Fig 3) with accepted model fit. This would represent the final measurement of the actual use of StaySafe PH among Filipinos. Moreover, the comprehensive assessment of the initial and final SEM factor loadings for evaluating the variables of intent to utilize the StaySafe PH application is given in Appendix S2 Table. The corresponding mean and standard deviation of the constructs of the variables were also included in the table.

The final model fit parameters are shown in Table 1. It can be observed that the parameter estimates after removing certain factors and relationships have passed the minimum cut-off. Six measures were considered for the goodness of fit, which attained the minimum cut-off by having a value greater than 0.80 [101]. The Root Mean Square Error (RMSEA) met the minimum cut-off by having a value less than 0.07 [102]. The final model has passed the suggested cut-off. Thus, it can be implied that the model has improved and can be an adequate representation of the population.

**Table 1:** Model fit parameters.

Goodness of fit measures of SEM	Parameter Estimates	Minimum cut-off	Suggested by
Incremental Fit Index (IFI)	0.889	>0.80	Gefen et al. [101]
Tucker Lewis Index (TLI)	0.882	>0.80	Gefen et al. [101]
Comparative Fit Index (CFI)	0.898	>0.80	Gefen et al. [101]
Goodness of Fit Index (GFI)	0.859	>0.80	Gefen et al. [101]
Adjusted Goodness of Fit Index (AGFI)	0.829	>0.80	Gefen et al. [101]
Root Mean Square Error (RMSEA)	0.062	<0.07	Steiger [102]

The composite reliability and validity of the latent variables are shown in Table 2. The Cronbach alpha and Composite reliability determine the reliability of the indicators used to reflect on the factors. Thus, the higher the reliability, the more internal consistency in the indices [103]. The usual acceptable range for the Cronbach Alpha and Composite Reliability is 0.60 to 0.70 [104]. Moreover, the average variance extracted (AVE) has values greater than the threshold (> 0.50) [37, 66]. Thus, there is internal validity and reliability. The overall direct, indirect, and total effects of the relationships of the latent variables, with the respective p-values are presented in the appendix section (S3 Table).

**Table 2:** Composite reliability and validity.

Factor	Cronbach’s α	Composite Reliability (CR)	Average Variance Extracted (AVE)
Understanding the COVID-19 Vaccine	0.949	0.951	0.795
Understanding the COVID-19 Delta Variant	0.926	0.925	0.713
Perceived Vulnerability	0.823	0.835	0.562
Perceived Severity	0.899	0.904	0.656
Performance Expectancy	0.965	0.959	0.822
Social Influence	0.958	0.959	0.823
Hedonic Motivation	0.979	0.976	0.891
Behavioral Intention	0.958	0.952	0.800
Actual Use	0.968	0.966	0.850
System Usability Scale	0.944	0.946	0.639

### 4.2 Deep Learning Neural Network

DLNN was performed by doing ten iterations for each combination with 150 epochs [105]. The number of nodes for the hidden layer used in this study is from 10 to 100. Thus, a total of 19,800 runs for the initial optimization of the data were done. The summary of the initial optimization is presented in Table 3.

**Table 3:** Summary of initial DLNN run.

Latent	Node H	AF H	AF O	Optimizer	Average Train	StDev	Average Test	StDev
UDV	70	swish	softmax	adam	75.308	4.709	66.164	3.170
UV	50	tanh	sigmoid	adam	80.750	3.375	64.923	2.812
PV	40	swish	sigmoid	adam	79.357	3.895	65.923	4.245
PS	100	tanh	sigmoid	adam	77.774	3.943	64.538	5.863
PE	30	tanh	sigmoid	RMSProp	89.959	8.121	65.003	4.153
EE	50	swish	sigmoid	adam	78.339	4.613	65.231	4.541
SI	30	swish	sigmoid	adam	78.903	5.853	65.923	4.784
HM	90	swish	sigmoid	adam	74.678	3.624	64.715	4.599
HB	80	tanh	softmax	adam	78.284	3.957	65.462	3.740
BI	70	tanh	sigmoid	adam	76.742	4.641	65.462	4.579
AU	100	swish	softmax	adam	75.846	4.475	64.924	2.789

The study utilized Analysis of Variance (ANOVA) to determine the factors that have the highest output and parameters, leading to the best combination. Based on the results, UDV had the highest training and testing ratios with 66.16% and a standard deviation of 3.170. The parameters with the best output for UDV were Swish as the hidden layer with 70 nodes, Softmax as the output layer, and Adam as the optimizer. Moreover, the highest accuracy with its parameters was considered for the final optimization.

**Fig 4:**
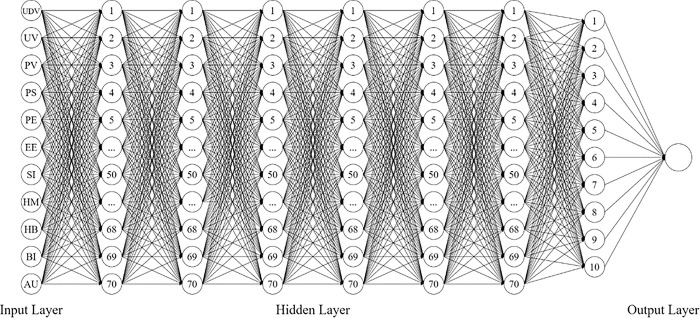
Deep neural network.

The final optimization of DLNN considered the number of hidden layers, considering 5, 6, and 7 with 70 nodes, 70:30 and 80:20 training and testing ratio, and was run with 200 epochs. Results showed the highest average accuracy of 92.32% with 3.641 standard deviations for 6 hidden layers with 70 nodes at an 80:20 training and testing ratio. UDV was seen to be the most significant factor contributing to the intention to use the contact tracing application, consistent with the results of the SEM. Presented in Fig 4 is the optimum DLNN for predicting the factors affecting the intention to use a contact tracing application, StaySafe PH among Filipinos.

### 4.3 System Usability Scale

The SUS was utilized in this study to determine the usability of the StaySafe PH application among Filipinos. The statistical analysis of the SUS is presented in Table 4. It can be observed that the minimum SUS score is 0 and the maximum is 100. The result of the average SUS score is 64.39, with a standard deviation of 19.29. The SUS score got a grade of D, which means the usability of the StaySafe PH application is poor. Given the results, it can be implied that the Filipinos deemed the usability system of the application to be substandard and needed improvement. To investigate further, the reviews of the application in Google Play are 3.1 stars out of 5 and 1.9 stars in the AppStore. The user’s feedback mainly was about how it is not user-friendly, and the application is too complicated. Mayor Magalong of Baguio City has also said that the application is not highly reliable for their LGU’s official contact tracing application. The developers should enhance the application’s functionalities [106].

**Table 4:** Statistical analysis of the SUS.

**Mean**	64.3885
**Standard Deviation**	19.2946
**Minimum Value**	0
**Maximum Value**	100
**Range**	100

Table 5 presents the number of respondents who graded the system’s usability A, B, C, D, and F. Its corresponding interpretation is also observed in the table. The results show that most of the respondents graded the usability system of the StaySafe PH application F, which was interpreted as Awful. The second highest is a grade of D, which means Poor. The third highest is a grade of A, which corresponds to Excellent, and the fourth is B which means Good. Lastly, there is 0 respondent who graded the StaySafe PH application C, which conforms to Okay.

**Table 5:** SUS grade and corresponding quantity.

SUS Score	Grade	Quantity	Adjective
>80	A	127	Excellent
68–80.3	B	120	Good
68	C	0	Okay
51–68	D	140	Poor
<51	F	259	Awful

## 5. Discussion

The SEM and DLNN presented similar findings, indicating that Understanding the COVID-19 Delta Variant (UDV) has the highest significant effect on Perceived Vulnerability (PV) (β = 0.193, P = 0.009) and has a significant direct effect on Perceived Severity (PS) (β = 0.582, P = 0.020). Based on the results, being educated on the COVID-19 Delta variant, how it mutates, transmitted, and its corresponding safety precautions positively affects the PS and PV. It can be implied that individuals perceive that they are vulnerable and can be severely affected by the virus because they have adequate knowledge regarding the Delta variant. According to Superio et al. [107], information plays a big role in people on how they would view the diseases and how risky it is to their health. Thus, the more educated and understood the Delta variant, the more people were able to know how vulnerable and severe the effects of the COVID-19 Delta Variant were. In this case, UDV has the most significant effect on PS than PV. In the study of Mant et al. [108], individuals are most likely to learn that COVID-19 cases have decreased in some countries. Thus, it affects how they perceive the severity of the virus. Compared to the study of Chow et al. [14], people in Singapore did not have any problems on the uptake for their contact tracing application. That is, the trust provided significance on the inclination in using the application rather than understanding the impact first. With the evident proliferation of the COVID-19 virus before, Singaporean have had a quick response on the contact tracing application consideration. It can be implied that Filipinos may learn that the pandemic situation in some countries is not difficult, thus making them feel that the pandemic effects are less severe.

Understanding the COVID-19 Vaccine (UV) has been found to have a significant effect on PS (β = 0.508, P = 0.028) and a significant correlation with UDV (β = 0.825). Knowledge of how the COVID-19 vaccine works, its corresponding side effects and its importance are significant to the PS. Even though the vaccine helps in averting the effects of COVID-19, the individuals still know the dangers of COVID-19 and can perceive that the effects are severe to their health, but they are not vulnerable, as implied by the SEM results. COVID-19 vaccines can greatly help to control the pandemic. However, it does not fully stop COVID-19 from entering the system. Thus, it only reduces its effects on the individual. In this case, there is still a need to be alarmed and follow the safety precautions [109, 110]. Contrary to the findings of Khan et al. [12], generic highlights were on the use of information obtained from the COVID-19 tracing application from the vaccine passports. It was dealt with negative notion as information, security, and data privacy were on the line. The results in this study can also suggest that individuals in the Philippines expected and were certain that vaccination would help them not contract the virus and thus not be vulnerable, leading to a positive actual use of the application. Therefore, they ought to know that they are immune enough to the effects of COVID-19 for them not to be vulnerable.

PV based on the results has a significant effect on Actual Use (AU) (β = 0.341, P = 0.003). Individuals were most likely to use the StaySafe PH application as a preventive measure in combating COVID-19. This was because they perceived themselves as vulnerable to contracting the virus. It was indicated that people who are facing health threats would promote an action to protect themselves [111]. It was added that they would consider all actions to avoid health risks. On the other hand, PS has a significant negative effect on AU (β = -0.265, P = 0.016). Based on the SEM, it could be posited that even if the respondents deemed the threats severe, they would not use the application. In the study of Stangier et al. [112], individuals with high levels of PV to diseases have a high adaptive behavior and would participate in any preventive measures. In relation to the study of Tomczyk et al. [113], people who perceive enjoyment in using the application would promote its use. However, social influence and perceived ease of use among contact tracing applications presented low significance in their study. It could be posited that a person’s own attitude would promote the use of these types of applications. Similarly, Kwarteng et al. [114] presented similar findings and posited that personal norms, barriers, willingness, and security prompted significance. However, both social influence and behavioral control did not show significant effects.

Moreover, Performance Expectancy (PE) has a significant impact on Behavioral Intention (BI) (β = 0.489, P = 0.006). The results implied that individuals deemed that they would use the StaySafe application because it has a lot of benefits and lives up to their expectations which will be useful in their life to prevent themselves from contracting the virus. PE was among of the vital elements that impacted the BI in the study [115]. It is believed that the consumers would intend to use an application that has what they need. Filipinos value the benefits that they can get in using the application, which would protect themselves from harm, synonymously to the study by Zhu et al. [116]. The application’s main features include alerting you when there is a positive patient within your vicinity. Thus, Filipinos know when they are being exposed to the virus. The application also shows updated data on COVID-19 cases, recoveries, and deaths [24]. This is similar with other countries as the COVID-19 virus was evident and may target any people [117]. Therefore, the Filipinos consider the StaySafe application to help evaluate the risk of exposure to the COVID-19 Delta variant. Given the application’s features, the Filipinos observed and believed that the application was helpful in their everyday life. It also increases their chance of being safe and getting things done quickly. The application’s merits persuaded people to utilize it. In the study by Walrave et al. [39], the desire to utilize the COVID-19 contact monitoring program was best predicted by PE.

Societal impact has also been demonstrated to have a substantial influence on BI (β = 0.241, P = 0.016), as well as Hedonic Motivation (β = 0.249, P = 0.007). The results of SEM suggested that the individuals value the suggestions and opinions of their family, peers, and professionals which led them to intend to use the StaySafe application. Individuals have an intention to use and install the StaySafe PH application because the application is enjoyable to use, and it is entertaining for the users. The results and claims are also aligned with the study of Chan et al. [118], which indicated that people who are important to the individual would influence them to use a suggested technology. The determination of utilizing the application is heavily influenced by societal pressures. In the study by Fadzil [115], it was stated that entertaining applications would greatly affect the people to use an application. The higher the HM towards new technology, the more the individuals will have a motivation to utilize the program [62]. Contrary to the findings, Chambers et al. [10] implicated that people utilized the application due to its efficient and fast transaction. HM was not measured as enjoyment was not one of the primary reasons for using the application, rather, health and safety concerns. On the other hand, the outcome matches up with the research of Alam et al. [41] that utilizes the SEM-ANN approach and found that the HM is a significant factor in adopting mobile health applications.

BI has also been shown to have a large consequential impact on the AU (β = 0.249, P = 0.007). The results imply that the respondents would use the StaySafe application when they have an intrinsic intention to use it. The relationship between BI and AU can be justified by the claims of Alam et al. [41], who mentioned that individuals would transform from the coherent stage to the action stage. According to the study by Raman and Don [119], the higher the behavioral intention of the individuals, the higher the chances that they would use the application. Consequently, SUS has shown a significant direct effect on the AU (β = 0.292, P = 0.004). Based on the results, the respondents deemed that SUS influences the AU since as they are using the application, the usability aspect of the StaySafe application should matter and is an important factor. A usable system can aid in the overall effectiveness of an application. Thus, using a contact tracing application, specifically the StaySafe application, should be user-friendly to aim for its maximum effectiveness [120].

From the findings, several factors showed insignificant effects. Effort Expectancy has revealed that it has an insignificant relationship with BI. Based on the results, the respondents are mostly made up of 15 to 24 years old (47.21%) and 25 to 34 years old (21.83%). According to the study by Chan et al. [118], they are the age groups that are literate with new technology and do not require effort in trying new applications such as the contact tracing application. Thus, it implies that convenience does not influence the intention to use the StaySafe application. The results are also the same as the study of Fadzil [115]. However, other studies have provided significance on the effort expectancy variable on their contact tracing application [Gerli et al. [33] Li et al. [28]].

In addition, Habit was also found to have no significant direct effect on the BI. Consequently, the results contradict the studies of Cheng et al. [121] and Ramírez-Correa et al. [122] as they mentioned that Habit greatly influences the intention to use a mobile application. It can be implied that the respondents only use the StaySafe PH application when required by the establishments and for that sole purpose only. Based on the demographics, several respondents (31.27%) do not use the StaySafe application, which could be the sole reason for the insignificance of Habit to BI. The findings are consistent with the research conducted by Raman and Don [119], the insignificance of Habit may be due to the application not being made to a requirement to use. However, studies like that from Chambers et al. [10] implied greater response on the uptake of contact tracing application due to its ease among media comments and tweets. This means that proper design is needed for actual use and positive utility among people, which the StaySafe PH may need further development as evidenced by the SUS output.

### 5.1 System Usability Scale

In utilizing the SUS, the perceived usability of the StaySafe PH application was determined. This was considered as a variable (changing the measure items as all positive to align with the SEM analysis), and a scale when assessing the technology. To which, the application garnered a grade of D, which is equivalent to the "Poor" usability of the application. The application should have improved for the Filipinos to be able to use the application with ease. The contact monitoring software is required for impact regulation of COVID-19. Thus, the contact tracking application’s functionality plays a significant role for all people to use an application with ease [64]. The more people use the contact tracing application; the more influential the contact tracing would be [74, 123].

According to Hägglund and Scandurra [124], the SUS is not capable of determining the specific problems of the system or its effectiveness and efficiency. It can only aid in defining and understanding the system’s usability level. Thus, the results suggested that the application should fix and revise its user interface. Based on their feedback in Google Play and AppStore, users mentioned that the application was too confusing for them. This is one of the main reason for the negative uptake and utility of the application. Therefore, it is suggested that better navigation, interface, and overall system design should be observed so people could consider it. The application should therefore have a neat layout that will be easier for the users to use and install [125]. In addition, the application may also consider having guidelines or step-by-step procedures for utilizing the application. Further studies that apply comprehensive usability testing and evaluation could be done to know the specific issues and address them directly. The redevelopment, assessment, and redesign should be considered for future use of the application in the unforeseen health circumstances. This would help in the promotion of health monitoring, assessment, and proliferation of information in the future.

### 5.2 Theoretical implications

Several studies use the Protection Motivation Theory, Unified Theory of Acceptance and Use of Technology 2, and the System Usability Scale. There are also instances wherein some researchers combine the PMT and the UTAUT2 in new technology but not in a mobile application. This study specifically employed the three theories together, PMT, UTAUT2, and the SUS. In the study by Zhu et al. [116], it was additionally discovered that Perceived Vulnerability possesses a substantial beneficial link towards the health application for the young group whenever there is a new health threat. They deem themselves vulnerable, and it creates pressure for them to try new things to protect themselves, thus being more willing to use technology that is helpful and efficient. On the other hand, Actual Use of the UTAUT2 has been found to have a significant relationship with the SUS. There has been no study found on which the UTAUT2 and the SUS have integrated.

The integrated models of PMT, UTAUT2, and the SUS used in this work might lead to new conceptual considerations in mobile software and new technologies, which could generate benefits in the fields of health, disaster, etc. The PMT focuses on the protective behavior of individuals based on the acquired threats and danger [126]. The study revealed that the PMT factors have a significant relationship with the AU. Thus, individuals will resort to using the application if a threat would harm them. The UTAUT2 is an effective tool to determine the people’s willingness to utilize and adapt modern technologies [61]. Correspondingly, in the study, several factors of UTAUT2 have been found to have a significant direct relationship with the BI. Thus, implying that the UTAUT2 is a successful model in determining the influences on the decision to utilize the contact tracking software. The SUS has greatly helped the study by defining the StaySafe PH application level of usability and determining if the SUS affects the Filipinos’ motivation in employing the contact tracing application. Therefore, the integrated models could be extended and applied to other studies to know the intention to use an application that focuses on protecting individuals.

### 5.3 Practical implications

The results and findings of the study can help Filipino citizens by implementing a proper plan to assist in averting the effects of COVID-19. Part of the plan is to make the contact tracing application obligatory in all malls, establishments, and restaurants since the only way for the application to be practical and by making sure that almost everybody uses it. The majority of people should use a functioning and practical contact tracing application. Thus, the people’s trust in the application should play a significant role in its overall effectiveness [4]. The result also stated that 68.73% of Filipinos were only using the application, which is relatively low for an application that is necessary for the protection of their health. Social Influence has been found to have a significant effect, thus having an expert who would spread awareness of the importance of the contact tracing application would greatly help make the Filipinos use it to protect themselves from the danger further. It could be implicated that large concerns stem from the trust issues on privacy, data security, and overall consent in the information needed. This is one area that should be promoted. That is, how the data is stored and kept, utilized, and covered by the system, how the assurance could be provided among consumers, and how the government would manage the information obtained. Building the trust is crucial on the development and deployment of these types of technology.

Providing better and adequate knowledge for the people would also be a great help to the government. Based on the results, there seems to be a strong connection among Understanding the COVID-19 Delta Variant and the COVID-19 Vaccine to the Perceived Severity and Perceived Vulnerability. Thus, the more they are aware of and understand the situation, the more they will know what kind of threats they are about to face when the danger comes. Lack of knowledge can be a barrier to knowing the importance of a preventive measure. Therefore, knowledge is essential to understand further the importance of a health-protective device or application [127]. Bringing awareness to those people, especially those who are not well informed will help them know the importance of the preventive measures protocols and resort to using them daily. It is also vital for the government to relay information in layman’s terms for the people to understand and comprehend despite their educational background.

The developer of the StaySafe application may apply the research’s insights to incorporate a better system in their application as they also received several backlashes from users because of how the application is not helpful and does not serve its purpose [19]. The developers could also make the application more amusing to use. As stated in the results, Filipino users would likely engage in the fun and enjoyable application, thus, making the application enjoyable would yield more users using the application as the Hedonic Motivation profoundly impacts the motivation to utilize the StaySafe Philippines. Concentrating toward improving the benefits of the application because, in the results, the Performance Expectancy directly affects the behavioral intention; thus, when the application is deemed to be more helpful to the users, they would install and use it in their daily lives.

### 5.4 Limitations and future research direction

This investigation contains multiple drawbacks that might be addressed in subsequent research. Initially, the study is only limited to the Philippines setting. Thus, the respondents are Filipinos residing in the Philippines. Focusing on a particular community, province, or city can be an excellent addition to future research as each place has its own culture and way of life, affecting its intention to employ an app for tracking contacts. Second, the StaySafe PH program is the main subject of this study since it is the official contact tracing application. Thus, a different contact tracing application that is available and also used in the Philippines such as Traze and SafePass, can focus on future studies. The assessment of other available contact tracing applications may provide other results, which can concurrently be integrated to create a generalized pattern for contact tracing in the Philippines and even in other countries. Third, the authors surveyed through an online survey which those with internet access would most likely answer, such as the working population [128]. Disseminating the survey questionnaires traditionally would yield to older age groups can be the method of succeeding studies, which would help generate more represented results for the Philippines. In addition, the utilization of interviews may result in factors that may have not been considered in this study. Lastly, this study focused on a specific population in the Philippines, which provides deep insights within that context. However, considering the universal challenge posed by COVID-19, including a broader demographic analysis could enrich the study. This might involve stratifying data by different age groups, socio-economic statuses, or even including rural versus urban populations to explore whether and how these segments differ in their response to technology adoption.

## 6. Conclusion

Contact tracing is a component amongst precautionary procedures used to combat the spread of the COVID-19 virus. Several research investigations have been conducted throughout the world concerning an individual’s adoption or desire to utilize their country’s official contact tracing. However, little to no research has been conducted with the purpose of using the Philippines’ authorized contact tracking system, StaySafe PH. Therefore, the author wanted to investigate the elements that influence Filipinos’ willingness to adopt StaySafe PH integrating the PMT, UTAUT2, and the SUS utilizing the SEM and DLNN approach. The SUS was also used to determine the application’s level of usability. In utilizing the SEM and DLNN, the highest significant factor for the intention to use the StaySafe PH was the Understanding of the COVID-19 Delta Variant. Based on the SEM, Understanding the COVID-19 Vaccine, Understanding the COVID-19 Delta Variant, Perceived Vulnerability, Perceived Severity, Performance Expectancy, Social Influence, Hedonic Motivation, Behavioral Intention, Actual Use, and System Usability Scale are the significant factors affecting Filipinos’ intention to use on StaySafe PH application.

The SUS results have revealed that the application still needs improvement with its user interface. The StaySafe PH application got a score of "D," which corresponds to a poor usability system. It could be implicated that large concerns stem from the trust issues on privacy, data security, and overall consent in the information needed. This is one area that should be promoted. That is, how the data is stored and kept, utilized, and covered by the system, how the assurance could be provided among consumers, and how the government would manage the information obtained. Building the trust is crucial on the development and deployment of these types of technology. In addition, the application may also consider having guidelines or step-by-step procedures for utilizing the application. Further studies that apply comprehensive usability testing and evaluation could be done to know the specific issues and address them directly. The redevelopment, assessment, and redesign should be considered for future use of the application in the unforeseen health circumstances. This would help in the promotion of health monitoring, assessment, and proliferation of information in the future.

Overall, the study determined the elements that have a substantial impact on the motivation of utilizing the application, which are UV, UDV, PV, PS, PE, SI, HM, BI, AU, and SUS. The UDV was shown to have a particularly pertinent influence, implying that understanding the COVID-19 Delta variant can be the determining factor in using the application. Knowing about it can mean knowing its effects and thus knowing the preventive measures to prevent the threat. The study suggested that the application should be improved by making the user interface user-friendly for all ages and more effective. The results in this study can also suggest that individuals in the Philippines expected and were certain that vaccination would help them not contract the virus and thus not be vulnerable, leading to a positive actual use of the application. The government should impose effective communication and education about COVID-19 to make individuals knowledgeable about the pandemic and prevent further spread. This study has aided in the analysis of the motivation to utilize contact monitoring programs as well as investigated the integration of PMT, UTAUT2, and SUS in a framework. Furthermore, it is suggested that subsequent investigations extend and use the concept regarding contact tracing applications or technology adaptation in other countries.

## Supporting information

S1 Table Demographics. (DOCX)

S2 Table Indicators statistical analysis. (DOCX)

S3 Table Direct, indirect, and total effects. (DOCX)

## List of abbreviations

PMT: Protection Motivation Theory
UTAUT2: Unified Theory of Acceptance and Use of Technology
SUS: System Usability Scale
SEM: Structural Equation Modeling
DLNN: Deep Learning Neural Network
UV: Understanding COVID-19 vaccine
UDV: Understanding COVID-19 Delta Variant
PV: Perceived Vulnerability
PS: Perceived Severity
PE: Performance Expectancy
EE: Effort Expectancy
HB: Habit
HM: Hedonic Motivation
SI: Social Influence
BI: Behavioral Intention
AU: Actual Use
