# Supplementary material for: Factors affecting the intention to use COVID-19 contact tracing application “StaySafe PH”: Integrating protection motivation theory, UTAUT2, and system usability theory
Source: PLoS One. 2024 Aug 1;19(8):e0306701. doi: 10.1371/journal.pone.0306701 (PMC11293755; doi:10.1371/journal.pone.0306701)
Supplement: S1 Table — (DOCX) [file pone.0306701.s001.docx]

Table A1. Demographics.

| **Characteristics** | **Category** | **N=646** | **Percentage** |
| --- | --- | --- | --- |
| Age | 15 to 24 years old | 305 | 47.21% |
|  | 25 to 34 years old | 141 | 21.83% |
|  | 35 to 44 years old | 109 | 16.87% |
|  | 45 to 54 years old | 50 | 7.74% |
|  | 55 to 64 years old | 25 | 3.87% |
|  | 65 years old and above | 16 | 2.48% |
| Gender | Male | 280 | 43.34% |
|  | Female | 366 | 56.66% |
| Province/City of Residence | Quezon City | 239 | 37.00% |
|  | Paranaque City | 60 | 9.29% |
|  | Manila City | 31 | 4.80% |
|  | Pasig City | 28 | 4.33% |
|  | Marikina City | 26 | 4.02% |
|  | Cavite | 25 | 3.87% |
|  | Taguig City | 19 | 2.94% |
|  | Muntinlupa City | 19 | 2.94% |
|  | Laguna | 17 | 2.63% |
|  | Rizal | 17 | 2.63% |
|  | Antipolo | 16 | 2.48% |
|  | Las pinas City | 14 | 2.17% |
|  | Makati City | 14 | 2.17% |
|  | Bulacan | 12 | 1.86% |
|  | Ilocos Sur | 12 | 1.86% |
|  | Caloocan City | 11 | 1.70% |
|  | Metro Manila | 11 | 1.70% |
|  | Pasay City | 7 | 1.08% |
|  | Mandaluyong City | 7 | 1.08% |
|  | San Juan City | 7 | 1.08% |
|  | Baguio City | 6 | 0.93% |
|  | La Union | 5 | 0.77% |
|  | Valenzuela City | 5 | 0.77% |
|  | Malabon City | 5 | 0.77% |
|  | Pangasinan | 5 | 0.77% |
|  | Navotas City | 4 | 0.62% |
|  | Pampanga | 4 | 0.62% |
|  | Tarlac | 3 | 0.46% |
|  | Bacolod | 3 | 0.46% |
|  | Batangas | 2 | 0.31% |
|  | Romblon | 1 | 0.15% |
|  | Davao | 1 | 0.15% |
|  | Nueva Vizcaya | 1 | 0.15% |
|  | Zamboanga city | 1 | 0.15% |
|  | camarines sur | 1 | 0.15% |
|  | Bataan | 1 | 0.15% |
|  | Isabela | 1 | 0.15% |
|  | Leyte | 1 | 0.15% |
|  | Maguindanao | 1 | 0.15% |
|  | Iloilo | 1 | 0.15% |
|  | Negros Occidental | 1 | 0.15% |
|  | Sorsogon Province | 1 | 0.15% |
| Educational Attainment | Elementary Graduate | 1 | 0.15% |
|  | Secondary Graduate | 170 | 26.32% |
|  | Diploma/Trade Certificate | 70 | 10.84% |
|  | Bachelor's Degree Holder | 326 | 50.46% |
|  | Master's Degree Holder | 55 | 8.51% |
|  | PhD/Doctorate Degree Holder Graduate | 24 | 3.72% |
| Income or Allowance per Month | Below PHP 15,000 | 286 | 44.27% |
|  | PHP 15,001 - PHP 30, 000 | 159 | 24.61% |
|  | PHP 30,001 - PHP 45,000 | 65 | 10.06% |
|  | PHP 45,001 - PHP 60,000 | 53 | 8.20% |
|  | PHP 60,001 - PHP 75,000 | 31 | 4.80% |
|  | PHP 75,001 - PHP 90,000 | 31 | 4.80% |
|  | Above PHP 90,000 | 21 | 3.25% |
| Are you enrolled to PhilHealth? | Yes | 361 | 55.88% |
|  | No | 285 | 44.12% |
| Do you use StaySafe Application? | Yes | 444 | 68.73% |
|  | No | 202 | 31.27% |
