# Supplementary material for: Factors affecting the intention to use COVID-19 contact tracing application “StaySafe PH”: Integrating protection motivation theory, UTAUT2, and system usability theory
Source: PLoS One. 2024 Aug 1;19(8):e0306701. doi: 10.1371/journal.pone.0306701 (PMC11293755; doi:10.1371/journal.pone.0306701)
Supplement: S2 Table — (DOCX) [file pone.0306701.s002.docx]

Table A2. Indicators Statistical Analysis.

| **Variable** | **Item** | **Mean** | **StD** | **Factor Loading** | |
| --- | --- | --- | --- | --- | --- |
|  |  |  |  | **Initial** | **Final** |
| Understanding the COVID-19 Vaccine | UV1 | 4.3700 | 0.71156 | 0.825 | 0.798 |
|  | UV2 | 4.3978 | 0.68963 | 0.841 | 0.853 |
|  | UV3 | 4.4907 | 0.62437 | 0.943 | 0.946 |
|  | UV4 | 4.4938 | 0.62441 | 0.954 | 0.965 |
|  | UV5 | 4.4303 | 0.68919 | 0.871 | 0.847 |
| Understanding the COVID-19 Delta Variant | UDV1 | 4.2895 | 0.72909 | 0.821 | 0.840 |
|  | UDV2 | 4.3824 | 0.67348 | 0.850 | 0.894 |
|  | UDV3 | 4.1997 | 0.75044 | 0.829 | 0.833 |
|  | UDV4 | 4.2477 | 0.76793 | 0.879 | 0.795 |
|  | UDV5 | 4.3390 | 0.68963 | 0.854 | 0.834 |
| Perceived Vulnerability | PV1 | 4.2198 | 0.70082 | 0.760 | 0.768 |
|  | PV2 | 4.2074 | 0.70678 | 0.807 | 0.832 |
|  | PV3 | 4.0960 | 0.77764 | 0.737 | 0.727 |
|  | PV4 | 3.3978 | 1.23059 | 0.295 | - |
|  | PV5 | 3.8808 | 0.92159 | 0.605 | 0.592 |
| Perceived Severity | PS1 | 4.2554 | 0.66689 | 0.816 | 0.763 |
|  | PS2 | 4.3282 | 0.65228 | 0.924 | 0.899 |
|  | PS3 | 4.3328 | 0.69040 | 0.808 | 0.808 |
|  | PS4 | 4.3189 | 0.66625 | 0.774 | 0.793 |
|  | PS5 | 4.2167 | 0.74673 | 0.598 | 0.639 |
| Performance Expectancy | PE1 | 3.9009 | 0.79305 | 0.909 | 0.931 |
|  | PE2 | 3.9427 | 0.76643 | 0.903 | 0.913 |
|  | PE3 | 3.8684 | 0.81822 | 0.931 | 0.911 |
|  | PE4 | 3.9133 | 0.81187 | 0.896 | 0.881 |
|  | PE5 | 3.8978 | 0.82712 | 0.894 | 0.869 |
| Social Influence | SI1 | 3.9211 | 0.83246 | 0.912 | 0.910 |
|  | SI2 | 4.0093 | 0.77554 | 0.897 | 0.885 |
|  | SI3 | 3.9567 | 0.80770 | 0.932 | 0.937 |
|  | SI4 | 3.9505 | 0.80542 | 0.949 | 0.951 |
|  | SI5 | 4.0387 | 0.75743 | 0.842 | 0.827 |
| Hedonic Motivation | HM1 | 3.4040 | 0.88532 | 0.923 | 0.925 |
|  | HM2 | 3.3901 | 0.86598 | 0.965 | 0.967 |
|  | HM3 | 3.3467 | 0.88250 | 0.960 | 0.961 |
|  | HM4 | 3.3638 | 0.87295 | 0.949 | 0.943 |
|  | HM5 | 3.3947 | 0.87546 | 0.951 | 0.945 |
| Habit | HB1 | 3.3406 | 0.99540 | 0.953 | - |
|  | HB2 | 3.3715 | 0.97792 | 0.967 | - |
|  | HB3 | 3.3947 | 0.96476 | 0.922 | - |
|  | HB4 | 3.0573 | 1.03496 | 0.732 | - |
|  | HB5 | 3.5867 | 0.94389 | 0.758 | - |
| Effort Expectancy | EE1 | 3.9876 | 0.79719 | 0.891 | - |
|  | EE2 | 3.9752 | 0.79104 | 0.909 | - |
|  | EE3 | 3.8994 | 0.81313 | 0.937 | - |
|  | EE4 | 3.9164 | 0.82545 | 0.937 | - |
|  | EE5 | 3.9071 | 0.82821 | 0.925 | - |
| Behavioral Intention | BI1 | 3.9381 | 0.80456 | 0.900 | 0.897 |
|  | BI2 | 4.0201 | 0.74785 | 0.899 | 0.879 |
|  | BI3 | 3.8808 | 0.83138 | 0.853 | 0.849 |
|  | BI4 | 3.9365 | 0.81116 | 0.881 | 0.891 |
|  | BI5 | 3.9628 | 0.80416 | 0.887 | 0.886 |
| Actual Use | AU1 | 3.2121 | 1.08545 | 0.922 | 0.922 |
|  | AU2 | 3.2508 | 1.07355 | 0.934 | 0.930 |
|  | AU3 | 3.3885 | 0.97684 | 0.855 | 0.858 |
|  | AU4 | 3.3003 | 1.04306 | 0.952 | 0.952 |
|  | AU5 | 3.2554 | 1.08325 | 0.946 | 0.947 |
| System Usability | SUS1 | 3.8545 | 0.82248 | 0.533 | 0.622 |
| Scale | SUS2 | 3.6099 | 0.85335 | 0.884 | 0.857 |
|  | SUS3 | 3.3498 | 1.10175 | 0.856 | 0.807 |
|  | SUS4 | 3.7817 | 0.85383 | 0.672 | 0.637 |
|  | SUS5 | 3.6316 | 0.85935 | 0.816 | 0.770 |
|  | SUS6 | 3.3251 | 1.04750 | 0.847 | 0.851 |
|  | SUS7 | 3.7136 | 0.90307 | 0.679 | 0.678 |
|  | SUS8 | 3.5681 | 0.90348 | 0.873 | 0.878 |
|  | SUS9 | 3.5232 | 0.96702 | 0.860 | 0.866 |
|  | SUS10 | 3.3978 | 1.09315 | 0.881 | 0.895 |
