# Supplementary material for: Factors affecting the intention to use COVID-19 contact tracing application “StaySafe PH”: Integrating protection motivation theory, UTAUT2, and system usability theory
Source: PLoS One. 2024 Aug 1;19(8):e0306701. doi: 10.1371/journal.pone.0306701 (PMC11293755; doi:10.1371/journal.pone.0306701)
Supplement: S3 Table — (DOCX) [file pone.0306701.s003.docx]

Table A3. Direct, Indirect, and Total Effects.

| **No** | **Variable** | **Direct Effect** | **P-Value** | **Indirect Effect** | **P-Value** | **Total Effect** | **P-Value** |
| --- | --- | --- | --- | --- | --- | --- | --- |
| 1 | UV → PS | 0.538 | 0.018 | - | - | 0.538 | 0.018 |
| 2 | HM → BI | 0.262 | 0.010 | - | - | 0.262 | 0.010 |
| 3 | SI → BI | 0.251 | 0.012 | - | - | 0.251 | 0.012 |
| 4 | PE → BI | 0.488 | 0.007 | - | - | 0.488 | 0.007 |
| 5 | UDV → PS | 0.256 | 0.003 | - | - | 0.256 | 0.003 |
| 6 | UDV → PV | 0.589 | 0.005 | - | - | 0.589 | 0.005 |
| 7 | BI → AU | 0.374 | 0.012 | - | - | 0.374 | 0.012 |
| 8 | PS → AU | -0.141 | 0.021 | - | - | -0.141 | 0.021 |
| 9 | PV → AU | -0.311 | 0.003 | - | - | -0.311 | 0.003 |
| 10 | AU → SUS | 0.458 | 0.006 | - | - | 0.458 | 0.006 |
| 11 | UV → AU | - | - | -0.076 | 0.016 | -0.076 | 0.016 |
| 12 | UV → SUS | - | - | -0.035 | 0.017 | -0.035 | 0.017 |
| 13 | HM → AU | - | - | 0.098 | 0.014 | 0.098 | 0.014 |
| 14 | HM → SUS | - | - | 0.045 | 0.018 | 0.045 | 0.018 |
| 15 | SI → AU | - | - | 0.094 | 0.005 | 0.094 | 0.005 |
| 16 | SI → SUS | - | - | 0.043 | 0.005 | 0.043 | 0.005 |
| 17 | PE → AU | - | - | 0.182 | 0.005 | 0.182 | 0.005 |
| 18 | PE → SUS | - | - | 0.0.83 | 0.004 | 0.0.83 | 0.004 |
| 19 | UDV → AU | - | - | 0.147 | 0.002 | 0.147 | 0.002 |
| 20 | UDV → SUS | - | - | 0.067 | 0.002 | 0.067 | 0.002 |
| 21 | BI → SUS | - | - | 0.171 | 0.013 | 0.171 | 0.013 |
| 22 | PS → SUS | - | - | -0.065 | 0.018 | -0.065 | 0.018 |
| 23 | PV → SUS | - | - | 0.142 | 0.003 | 0.142 | 0.003 |
